# Inflammatory aneurysm of the C1 segment of the internal carotid artery linked to ipsilateral internal carotid artery occlusion due to otitis media: A Case Report and Literature Review

**DOI:** 10.3389/fmed.2026.1902169

**Published:** 2026-08-25

**Authors:** Xufeng Sun, Zhifeng Wen

**Affiliations:** Department of Neurosurgery, The First Hospital of China Medical University, Shenyang, China

**Keywords:** otitis media, inflammatory aneurysm, internal carotid artery occlusion, inflammatory factors, Epstein–Barr virus (EBV)

## Abstract

**Background:**

Internal carotid artery occlusion (ICAO) is a very serious cerebrovascular disease, and ICAO caused by infection is extremely rare. We report a case of an extremely rare inflammatory aneurysm of the C1 segment of the ipsilateral internal carotid artery, caused by otitis media (OM), accompanied by ipsilateral ICAO.

**Case Presentation:**

A 59-year-old woman presented to the emergency department with a 40-day history of fever and headache, which had worsened over the past 30 days and was accompanied by slurred speech and numbness in the right side of her face. Upon admission, a head and neck CTA revealed near-total occlusion of the right internal carotid artery (ICA) at the C1 segment with a massive distal aneurysm. The patient was diagnosed with severe OM with secondary intracranial infection by laboratory results, medical history and physical examination; cerebrospinal fluid (CSF) analysis was positive for human herpesvirus type 4 (EBV). Following multidisciplinary consultation, the patient's symptoms improved with medication adjustment and treatment with antibiotics and antiviral agents. Follow-up CTA demonstrated resolution of the aneurysm and progression of the ICA to long-segment occlusion. No ischemic neurological deficits were noted. CSF analysis showed marked reduction in EBV viral titer. At 6 months post-discharge, there were no indications of recurrence or new symptoms observed.

**Conclusion:**

This case underscores an uncommon pathological route connecting OM, vasculitis, aneurysm development, and ICAO. The etiology and clinical progression of this ailment are exceedingly intricate; prompt identification and vigorous infection management are essential.

## 1 Introduction

Internal carotid artery occlusion (ICAO) is a grave cerebrovascular disease and a major cause of transient ischemic attacks (TIAs) and cerebral infarction. The annual stroke rates for asymptomatic and symptomatic ICAO are 4.4% and 6%−20%, respectively, with unilateral ICAO accounting for the vast majority of cases (1, 2). Clinically, the vast majority of patients present with chronic ICAO, because sufficient time allows for the establishment of collateral circulation, they often have no obvious symptoms or only mild symptoms. In contrast, acute ICAO can lead to malignant cerebral edema and a poor prognosis due to the failure of collateral circulation to develop in a timely manner. Otitis media (OM) is a common and prevalent disease that can significantly impair quality of life and lead to hearing loss. Based on duration of onset, it can be classified into acute and chronic forms. The causes of acute OM are numerous and include bacterial and viral infections, Eustachian tube dysfunction, allergies, physiological and pathological factors, and immunological factors, as well as environmental and genetic factors. The factors contributing to chronic OM remain unclear, but it is often characterized by ear discharge, hearing loss, and tympanic membrane perforation (3, 4).

The complications of OM are highly complex. Common extracranial complications include hearing loss, mastoiditis, subperiosteal abscess, facial paralysis, middle ear fistula, and developmental delay, while intracranial complications include various forms of meningitis, brain abscess, venous sinus thrombosis, hydrocephalus, cerebrospinal fluid leak, and encephalocele (5). ICA lesions caused by OM are rare and may result from erosive osteitis, thrombotic arteritis, or iatrogenic injury. They commonly manifest as ICA stenosis or cavernous sinus thrombophlebitis and less frequently as an aneurysm. However, inflammatory aneurysms caused by infection eroding the vascular wall represent the most lethal complication of otogenic intracranial infections. When an infection leads to the formation of an aneurysm in the ICA and is simultaneously complicated by complete occlusion of that artery, this coexisting pathological condition is extremely rare in clinical reports. We hereby report a rare case of an inflammatory aneurysm in the C1 segment of the ipsilateral internal carotid artery caused by OM, accompanied by ipsilateral ICAO, and discuss its pathogenesis, imaging characteristics, and management strategies.

## 2 Case report

A 59-year-old female was presented to the emergency department with the chief complaint of fever and headache for 40 days, which were aggravated in the last 30 days, associated with slurred speech and numbness of the right side of the face. Her symptoms were not yet adequately controlled and had not responded to two prior treatment courses. The patient has a history of diabetes, no smoking history, and no alcohol use.

### 2.1 Physical exam

Upon admission, the patient's temperature was 36.4 °C, pulse rate was 98 beats per minute, and blood pressure was 169/105 mmHg. The patient's respiration was steady, averaging 18 breaths per minute. Neurological examination: the patient was alert, cooperative during the physical examination, and had slurred speech. Ocular movements were full in all directions, with no nystagmus. Muscle strength in all four limbs was Grade V. Reflexes were normal in all four limbs, and no pathological signs were elicited. The patient had diminished pain sensation on the right side of the face; no definite abnormalities were found in the examination of pain sensation, light touch, motor sensation, position sense, or vibration sensation in the remaining areas. The finger-to-nose test was steady and accurate bilaterally, and the heel-to-knee-to-shin test was steady and accurate on both sides.

### 2.2 Laboratory examinations

Laboratory tests were completed; blood tests, renal function, and liver function were generally within normal ranges. Cerebrospinal fluid (CSF) analysis (PROT 794 mg/L; CL- 118 mmol/L; cell count 110 x 10^6^/L). The patient was negative for several infectious diseases including HIV and syphilis. Testing in the CSF for T-cells associated with tuberculosis and for antibodies to Toxoplasma, rubella virus, cytomegalovirus, and herpes simplex virus were all negative. The patient was also negative for a panel of rheumatic antibodies, antineutrophil cytoplasmic antibodies (ANCA) and antiphospholipid antibodies.

### 2.3 Imaging examinations

**Figure 1:**
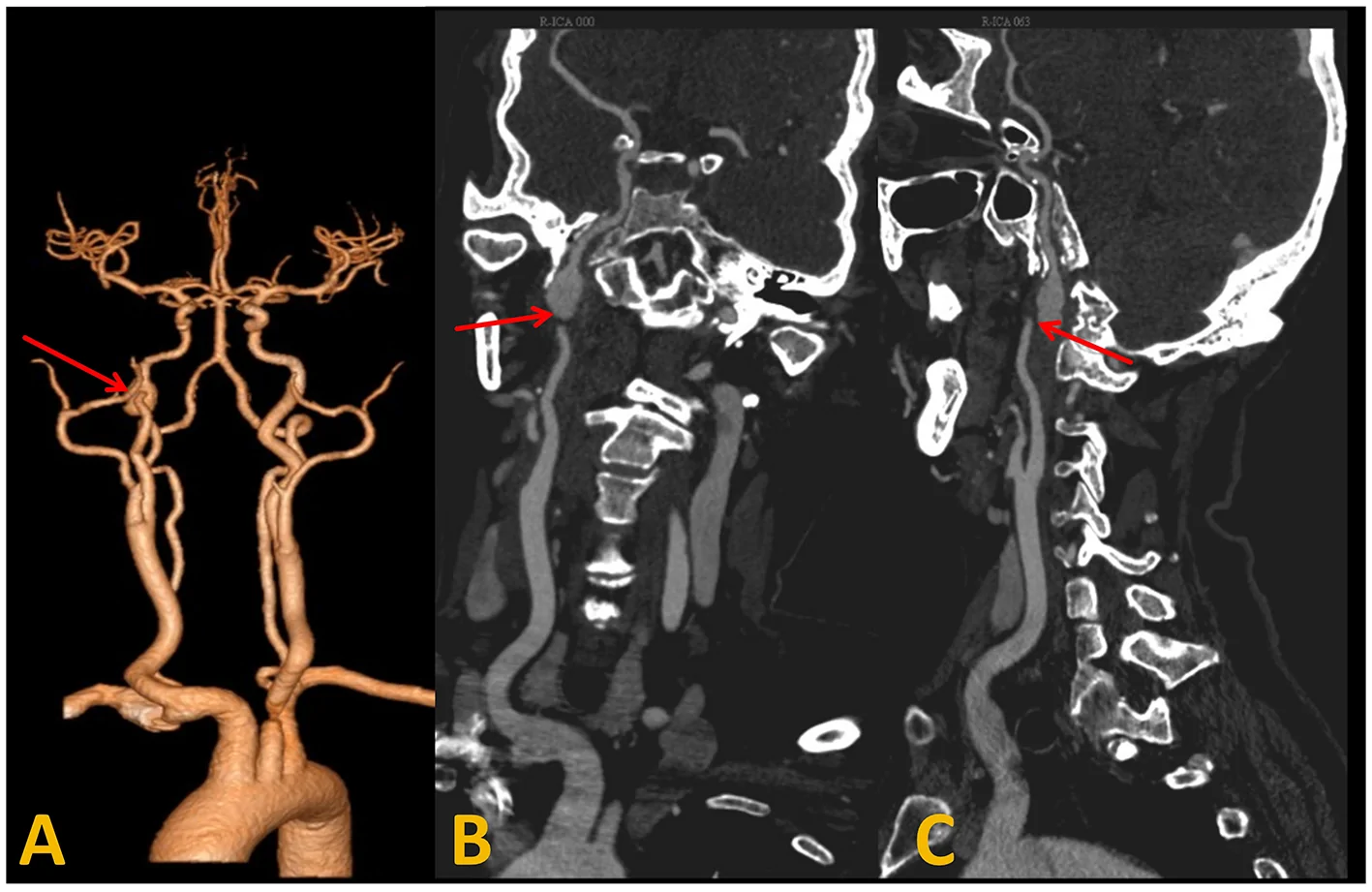
CTA of the head and neck on the day of admission. **A**, 3D-CTA; **B**, coronal 2D-CTA; and **C**, sagittal 2D-CTA of the head and neck taken on the second day of the patient's hospital stay. CTA indicates an aneurysm with severe proximal stenosis or near-total occlusion in the right internal carotid artery's C1–C2 segment.

Comprehensive imaging studies were performed, including head and neck CTA, uncontrasted and contrast-enhanced cranial MRI (MR+C), and pulmonary CT. The CTA revealed an aneurysm in the cervical segment of the right ICA, measuring approximately 1.3 cm, along with a suspected subtotal occlusion of the proximal ICA (Figure 1); MR+C revealed a distal inflammatory aneurysm of the cervical segment of the right ICA, accompanied by bone-enhancing lesions in the surrounding soft tissues and OM; pulmonary CT showed a nodular shadow of undetermined nature in the right lung.

### 2.4 Clinical course

After admission, the patient's condition worsened further and cerebral edema developed; mannitol and other medications were administered to reduce intracranial pressure. Pathogen detection was performed by metagenomic analysis of CSF and showed: DNA viruses: Human herpesvirus 4 (EBV); confidence: 99%; sequences: 298 Further serological screening for EBV antibodies revealed strongly positive results for EBV capsid antigen IgG and EBV nuclear antigen IgG and suspiciously positive results for EBV early antigen IgG and capsid antigen IgM. This indicates that a recent infection could have occurred. The CSF smear was negative for Cryptococcus. The patient presented with otorrhea. Endoscopic examination of the ear canal revealed no obvious abnormalities, and no signs of tympanic membrane perforation were observed. Given the complexity and severity of the condition, a multidisciplinary consultation was conducted on the fifth day of hospitalization. In accordance with the consultation recommendations, levofloxacin was replaced with piperacillin sodium and tazobactam sodium, and antiviral therapy with ganciclovir was initiated concurrently. Relevant tests and laboratory examinations were completed, and regular follow-up visits were scheduled.

**Figure 2:**
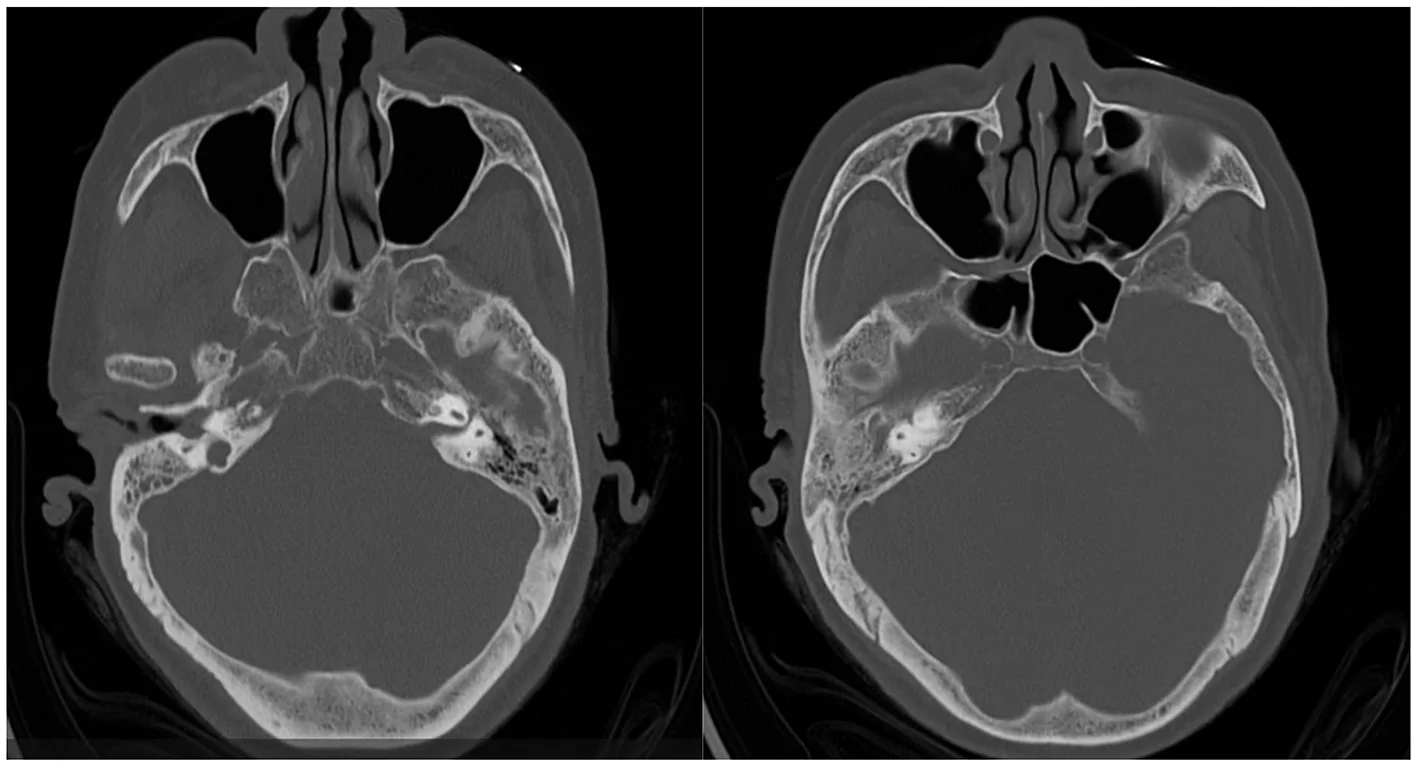
CT of the middle ear on the 6th day of hospitalization. Poor aeration of the right mastoid; areas of increased density are visible within the tympanic cavity and tympanic sinus. There is no obvious compression, displacement, erosion, or destruction of the ossicles; there is no enlargement of the tympanic cavity or tympanic sinus. No abnormal changes were observed in the inner ear structures, and no narrowing or dilation was noted in the external auditory canal.

**Figure 3:**
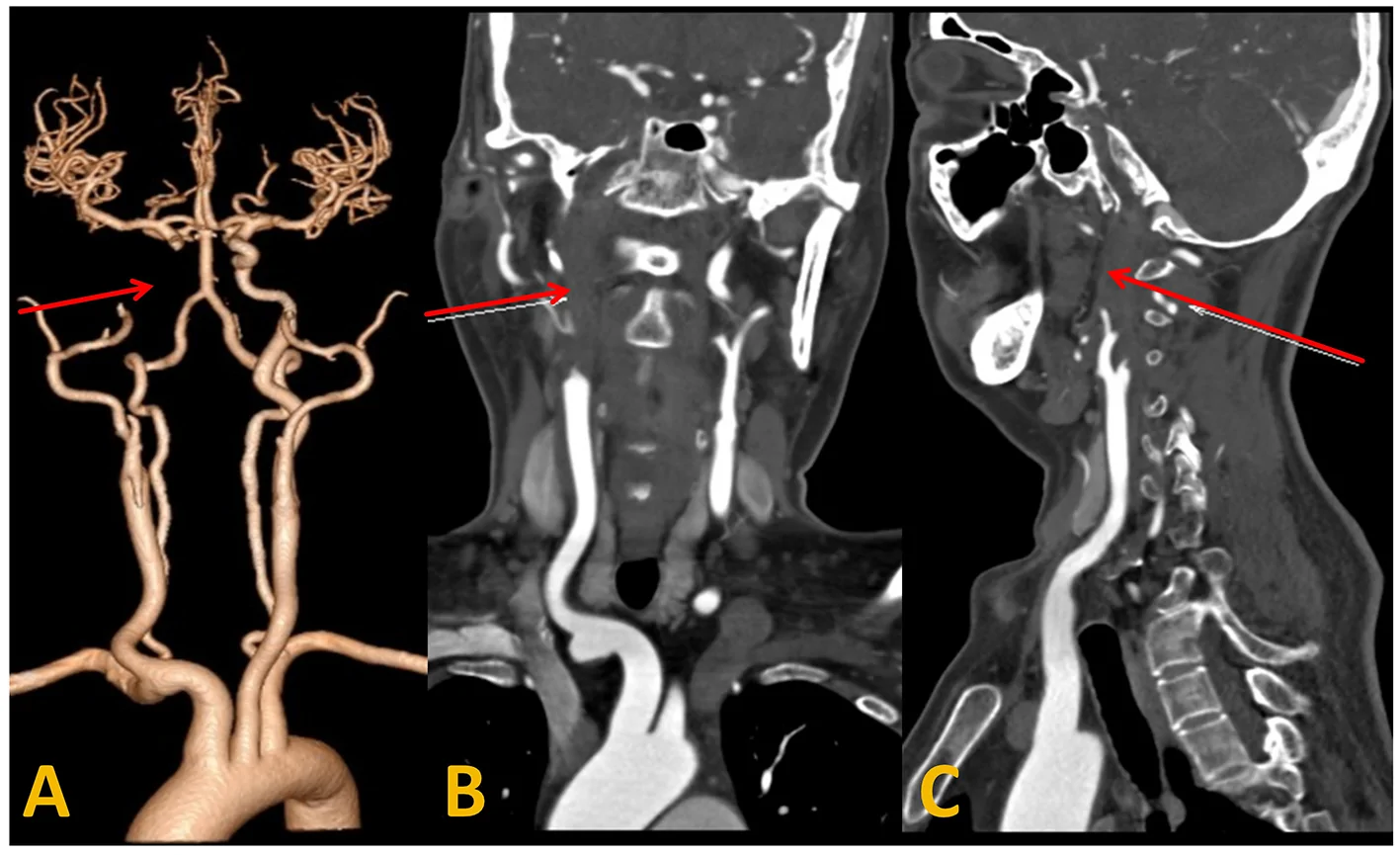
CTA reassessment conducted one month after patient admission. **(A)** 3D-CTA; **(B)** Coronal 2D-CTA; **(C)** Sagittal 2D-CTA. CTA demonstrates extensive occlusion of the right internal carotid artery, complete resolution of the aneurysm, patency of the anterior communicating artery, and satisfactory blood flow to the right middle cerebral artery and anterior cerebral artery.

On day 6 of hospitalization, the otolaryngology consultation after a CT (Figure 2) scan of the middle ear concluded that otologic surgery was not suitable in the context of the current severe infection and the presence of a massive aneurysm. Recommended treatment: oral medication and symptomatic treatment. On the 10th day of hospitalization, the patient's headache improved, but other symptoms persisted, so the treatment regimen was continued. By the third week of hospitalization, the patient's speech symptoms had improved and the sensation of numbness had diminished. Given that the patient's current condition permitted it, a lumbar puncture was performed to re-examine the CSF, which indicated a significant decrease in EBV titres. A follow-up lung CT scan performed during the fourth week of hospitalization showed a reduction in the size of the nodular shadows, and a follow-up MRI scan during the fifth week revealed a further reduction in the extent of the infection. However, during a follow-up CTA (Figure 3) 1 month later, we discovered right ICAO. A multidisciplinary consultation was therefore convened: although vascular bypass surgery is an option for ICAO, the patient's basilar circle blood flow was normal, indicating no corresponding clinical manifestations of occlusion. Additionally, since the current antimicrobial therapy was effective—with a recommended total treatment duration of 6–8 weeks—there were no indications for surgery at that time. Consequently, the current treatment regimen was continued. By the seventh week of hospitalization, the patient had largely recovered and reported no symptoms of discomfort, so he was discharged. At the six-month follow-up, the patient was in satisfactory condition with no new symptoms or recurrence. The patient's disease timeline is shown in Table 1.

**Table 1:** Patient's disease timeline.

Day	Event	Imaging	Lab	Treatment	Outcome
0	Fever headache	—	—	—	—
10	Worsening symptoms	—	—	Antibiotics	No improvement
14	Facial paralysis Hearing loss Dysarthria	—	—	Continue therapy	Symptom improvement
40	Worsening symptoms Admission	CTA: C1 aneurysm(+) near-occlusion	CSF: EBV+	Multidisciplinary management	Symptom improvement
40+ 5weeks	Follow-up	CTA: ICA occlusion, aneurysm resolved	CSF EBV ↓	Continue therapy	No ischemic deficits
40+6months	Follow-up	CTA: stable occlusion	—	—	No recurrence

## 3 Discussion

The adult middle ear primarily consists of the tympanic cavity and the Eustachian tube. Anatomically, the Eustachian tube is a short, complex, hourglass-shaped structure that plays a significant role in the surgical anatomy of the skull base. First, the posterosuperior portion of the Eustachian tube's bulge lies very close to the foramen ruptum. The inferior portion of the foramen ruptum is covered by fibrocartilaginous tissue, which firmly connects the ICA to the Eustachian tube. At the same time, the second crest of the ICA occupies the superior portion of the foramen ruptum; it is precisely through this fibrocartilaginous tissue that these structures are in direct contact with one another. Secondly, the bony portion of the Eustachian tube and the carotid canal are usually compartmented by a common wall, and in cross-section the first branch of the ICA is located immediately behind the bony- cartilaginous junction of the Eustachian tube (6). The petrous part of the ICA is in the petrous part of the temporal bone, and the external auditory canal is on the lateral surface of the temporal bone. Both structures are intimately related anatomically but separated by bone. The anatomical discrepancies or pathological states can change the relationship between the ICA and the external auditory canal, which is prone to hemorrhage, infection, and other complications.

Cervical segment aneurysms of the ICA are extremely rare, accounting for less than 1% of all ICA aneurysms. This segment is particularly prone to stenosis or dissection. Following the first report of a cervical segment ICA aneurysm resulting from trauma in 1975, research gradually began to explore the potential causes of aneurysms in the extracranial segment, including arterial dissection, surgical injury, fibromuscular dysplasia, tumor invasion, trauma, and inflammation (7, 8). Clinically, specific symptoms of cervical aneurysms may include headache, tinnitus, a pulsating mass, hoarseness, dysphagia, facial nerve palsy, bleeding from the ear or nose, hemorrhage due to rupture, and ischemic stroke; a tiny proportion of cases are accompanied by secondary ICAO (9, 10). Relevant literature indicates that the mortality rate for untreated extracranial ICA aneurysms can reach as high as 77%; even with surgical treatment, the mortality rate can reach 35%, often accompanied by permanent neurological deficits. Due to the fragility of the vessel wall and susceptibility to rupture, treatment is extremely challenging, and there is currently a lack of consistent clinical guidelines for diagnosis and treatment (11, 12).

In this article, we report a case of an inflammatory aneurysm of the cervical segment of the ICA caused by an OM infection, accompanied by secondary acute ipsilateral ICAO. We also further explore the mechanisms underlying the formation of this aneurysm and the secondary ICAO. Ischemic stroke caused by ICAO typically results in poor clinical outcomes and high mortality, which is often associated with inadequate collateral circulation. Although acute ICAO accounts for only 4.15%, the range of ICAO lesions is quite broad. Due to varying thrombus sizes and causes of occlusion, occlusion can occur anywhere from the origin to the terminal segment of the ICA and clinically may present with a spectrum of manifestations ranging from TIAs to severe ischemic strokes (13, 14). Most previously reported cases involved ICAO directly secondary to infection or inflammation, without mentioning the process that led to the formation of an inflammatory aneurysm.

In this case, the patient initially developed an inflammatory aneurysm resulting from an otitis media infection. Prior to admission, the infection may not have been effectively controlled, as evidenced by a massive fusiform aneurysm on the CTA. From a macro perspective, infectious inflammation plays a significant role in the formation, remodeling, progression, and eventual regression of aneurysms. Generally speaking, inflammation of the vascular wall involves events such as endothelial damage, inflammatory cell infiltration, extracellular matrix remodeling, thrombosis, and the complement cascade. Recent studies have shown that hemodynamic factors can also induce arterial wall damage and trigger wall inflammation and remodeling (15). From a microscopic perspective, the inflammatory response in aneurysms can be attributed to an imbalance between pro-inflammatory and anti-inflammatory factors. For example, interleukin-1β (IL-1β) is a multifunctional cytokine; its overexpression promotes lymphocyte activation, enhances immune cascades, and modulates inflammatory pathways. It can also damage the extracellular matrix and vascular smooth muscle cells (SMCs) and directly stimulate SMC apoptosis, leading to thinning of the aneurysms wall (16, 17). In contrast to pro-inflammatory factors, anti-inflammatory factors act as protective factors against Aneurysms. However, anti-inflammatory factors often require doses tens or even hundreds of times greater than pro-inflammatory factors to exert an inhibitory effect. Often, the effects of anti- inflammatory factors are offset by the multifunctionality of pro-inflammatory cytokines and the dominance of inflammatory cells, preventing them from fulfilling their function and consequently leading to the development and progression of aneurysms (18).

The patient subsequently experienced a phase in which the aneurysm resolved, followed by acute ICAO caused by inflammation. Clinically, common causes of acute ICAO include plaque rupture in carotid stenosis, cardioembolism, arterial dissection, moyamoya disease, autoimmune diseases, medications, vasculitis, and inflammatory infections. Among these, acute occlusion caused by inflammation is extremely rare in clinical reports; therefore, we consider that other factors may also be contributing to the ICAO. Before admission, the patient had pulsatile pain on the right side, numbness on the right side of the face, hearing loss in the right ear, and discharge of fluid from the right ear. These symptoms are consistent with OM and its complication, mastoiditis, and they were associated with facial nerve paralysis. After admission, MRI and CT scans showed foci of infection in the cranium and in the lungs; these lesions had markedly decreased at the time of discharge. Laboratory analysis showed that the patient's CSF contained EBV, but no other pathogen sequences were detected. Based on the patient's examination findings, laboratory results, and medical history, we hypothesized the mechanism underlying the formation of the aneurysm and occlusion. Initially, the patient's OM may not have been effectively controlled, leading to the spread of infection to the meninges and resulting in meningitis and a lung abscess. At the same time, the infection was spread hematogenously throughout the body, with initial damage of the endothelium, infiltration of inflammatory cells and release of inflammatory factors in the ICA. The infection then resulted in degradation of the extracellular matrix, reduced collagen fibers and SMCs apoptosis, ultimately resulting in thinning and remodeling of the vessel wall, abnormal hemodynamics and dilation of the vascular wall, creating an inflammatory aneurysm. Arterial stenosis has been reported to be associated with other viruses of the herpesvirus family. Similar to previous reports, this patient also experienced a marked change in EBV titers in the CSF. Since other types of viruses in the herpesvirus family have been reported to cause arterial stenosis, we therefore consider that the occlusion in this patient may be EBV-mediated.

ICA aneurysms caused by OM are relatively rare, but cases have been reported. Ryo Kusaka reported a case of a giant external segment ICA aneurysm caused by acute exudative OM; the aneurysm gradually resolved after flow-diverting stenting, but the ICAO observed in the present case was not reported in that study, which may suggest that our case is even rarer (9). We conducted a further review and compilation of the relevant literature, as shown in Table 2. None of the 11 patients with aneurysms caused by otitis media or mastoiditis reported in the literature had ICAO. Among them, 8 underwent endovascular treatment or bypass surgery; in 6 patients, the aneurysm eventually resolved or shrank; and only 1 patient died. Similar to the case reported here, the majority of these cases had favorable outcomes.

**Table 2:** Cases of inflammatory aneurysms associated with otitis media.

Year	Journal	Author	Age	Sex	Symptoms	Treatment	Outcome
2025	Journal of Neuroendovasc Ther	Ryo Kusaka et al.	57	Male	Facial paralysis	EVT	Aneurysm reduction
2022	The Journal of Laryngology and Otology	H Hyakusoku et al.	84	Famale	Otalgia	EVT	Symptom improvement
2018	Indian Journal of Otolaryngology and Head & Neck Surgery	Janez Mohorko et al.	60	Male	Meningitis	_	Die
2017	WorldNeurology	Yoichiro Kawamura et al.	32	Famale	Hearing loss Pulsatile tinnitus	Bypass grafting EVT	Aneurysm resolution
2015	Journal of NeuroInterventional Surgery	Justin R Mascitelli et al.	-	Famale	Otorrhagia	EVT	-
2013	BMJ Case&report	Alyssa S Shon et al.	79	Male	Otalgia	Mastoidectomy	Symptom improvement
2012	Pediatric Neurology	Senthilkumar Sankararaman et al.	0.5	Famale	Suppuration	-	Aneurysm resolution Internal carotid artery stenosis
2010	Neurologia medico-chirurgica	Hirofumi Oyama et al.	60	Famale	Otorrhagia	Bypass grafting	Internal carotid artery occlusion
2010	Neurologia medico-chirurgica	Mizuki Amano et al.	85	Famale	Vernet's syndrome	EVT	Aneurysm resolution
2006	Neurosurgery	Louis J Kim et al.	57	Famale	Otorrhagia	EVT	Aneurysm resolution
1996	No shinkei geka. Neurological surgery	K Kawakami et al.	67	Famale	Chronic otitis media	EVT	Aneurysm resolution

ICAO caused by infectious inflammation is most commonly associated with bacterial and fungal infections. A review of the relevant literature indicates that arterial occlusion caused by viral infections was extremely rare prior to the COVID-19 pandemic but has gradually increased since the onset of the pandemic; cases of unilateral or bilateral ICAO caused by coronaviruses have been reported. COVID-19 can lead to arterial thrombotic complications through inflammation, endothelial dysfunction, thrombin generation, and platelet activation pathways (19). Karan Seegobin also reported a case of coexisting hepatitis C virus infection and ICAO, although the specific mechanism remains unclear (20). Prior to 2019, most relevant reports involved human herpesvirus infections. Herpes simplex viruses (HSV-1 and HSV-2) are neurotropic viruses that can directly invade the vascular wall, causing inflammation and thrombosis (21). Rajesh Verma reported that herpes simplex virus type 3 (varicella-zoster virus) can cause arteritis leading to ICAO (22). HSV-4 (Epstein-Barr virus) and HSV-5 (cytomegalovirus) have also been cited in the literature as playing significant roles in atherosclerosis (23). Similarly, our case involved an ICAO associated with EBV infection. However, unlike the cases described in the aforementioned literature, this case involved the development of an aneurysm, a finding not previously reported in the literature.

We also explored a series of differential diagnoses. After admission, the patient underwent an external CSF test, which revealed no bacterial sequences; subsequent CSF cultures also showed no bacterial growth. Therefore, we first ruled out ICAO caused by a bacterial infection. During treatment, the patient underwent a series of laboratory tests, including a rheumatology panel, infectious disease panel, and anti-neutrophil cytoplasmic antibody (ANCA) testing; no positive results were found, so the likelihood of vasculitis was also considered low. Relevant imaging studies performed during treatment—including head and neck CTA, middle ear CT, and head MRI— revealed no obvious signs of vascular dissection; therefore, we also ruled out vascular dissection as a cause. The patient's examination and laboratory findings suggest a high likelihood of an intracranial inflammatory lesion accompanied by lymphadenopathy; combined with the clinical presentation, a neoplastic lesion is unlikely. The patient's echocardiogram showed no masses, and there was no history of cardiogenic stroke; therefore, the likelihood of occlusion caused by cardiogenic embolism is low. The patient also had no history of neck trauma or use of specific medications, and imaging studies did not reveal moyamoya disease; thus, these factors were also ruled out. The first CTA revealed severe stenosis or subtotal occlusion of the proximal C1 segment, though a small amount of blood flow was still present; distal to the stenosis, a massive inflammatory aneurysm was observed. Although the aneurysm had resolved by the time of the most recent CTA, the segment at the original aneurysm site and the distal segment were completely occluded—a condition that clinically often presents as a fatal stroke. Regarding the patient's absence of clinical symptoms of occlusion, we considered two factors: first, the patient's anterior communicating artery was patent, the circle of Willis was intact, and blood flow from the left ICA could supply sufficient perfusion to the right middle cerebral artery, anterior cerebral artery, and intracranial segments of the ICA. Second, the complete occlusion had lasted for over a month, during which time collateral circulation had sufficient time to develop; additionally, we administered appropriate medications to improve microcirculation and alleviate vasospasm. Therefore, it is possible that the patient has not suffered any significant sequelae of ischemia.

How should this challenging disease be diagnosed and treated? After reviewing the relevant literature, we have summarized the following points: (1) Regarding OM—the source of the condition—to reduce the incidence of complications, a multidisciplinary approach must be adopted at an early stage, including timely imaging and laboratory tests, as well as proper prevention and management under medical supervision. (2) For patients with symptomatic OM, drug therapy can be guided by laboratory test results until significant clinical, biological, and radiological improvement is observed. The optimal course of treatment should depend on the severity and extent of the disease, as well as the need for surgical intervention. (3) For patients with cerebral abscesses, surgical intervention may be considered. Surgical options primarily include supra- and retro-mastoidectomy to drain the abscess and resect areas of osteitis; less commonly, petrosectomy may be performed. Neurosurgical drainage of intracranial abscesses is generally not required, as the response to combined medical and otologic surgical treatment is relatively rapid (24, 25). (4) For inflammatory aneurysms, treatment for ICAO generally includes medical management and cerebral revascularization. However, the core issue in this case is inflammation. Based on our treatment plan and a review of the literature, we found that if an inflammatory aneurysm is not treated promptly and hemorrhage occurs, the risk of death is very high. Surgical options may include open surgery or endovascular treatment (EVT). (5) For ICAO, treatment options include antiplatelet therapy (aspirin and clopidogrel), surgical intervention (open surgery or endovascular treatment), avoidance of neck massage, maintenance of cerebral blood flow, and strict control of blood pressure and blood glucose levels. Since the patient in this case did not exhibit obvious symptoms of ICA ischemia during treatment, active surgical intervention was not performed.

## 4 Limitations

We have reorganized. This study also has some limitations. First, our sample size was small— consisting of a single case—and a review of the literature revealed few relevant reports of this type; therefore, our conclusions can only serve as a limited reference. Second, although the patient's entire diagnostic and treatment process was complete, considering potential future complications and disease progression, we should conduct long-term follow-up to determine the patient's ultimate clinical outcome. Third, our study primarily relied on a combination of imaging findings and clinical presentation. After careful analysis, we can only speculate that the Epstein-Barr virus may be the cause of ICAO, but we are unable to further validate this hypothesis, and the underlying pathogenesis remains unclear. To verify real-world outcomes, we should conduct basic experimental studies in the future to further substantiate our findings.

## 5 Conclusion

Infarction of the ICA resulting from infectious inflammation is rare. We present a case of an inflammation-induced aneurysm that subsequently led to obstruction. This patient developed an inflammatory aneurysm resulting from a history of acute OM, intracranial infection, and systemic infection, ultimately leading to ICAO. We hypothesize that the Epstein-Barr virus may play a central role in causing ICAO. Effective management of underlying diseases, swift prevention, and timely intervention for infectious inflammation, together with appropriate diagnostic assessments and medications, may be essential in the prevention and treatment of many problems.

## Data Availability

The original contributions presented in the study are included in the article/supplementary material, further inquiries can be directed to the corresponding author.
